# Effect of BDNF and Other Potential Survival Factors in Models of *In Vitro* Oxidative Stress on Adult Spinal Cord–Derived Neural Stem/Progenitor Cells

**DOI:** 10.1089/biores.2014.0058

**Published:** 2015-02-01

**Authors:** Laureen D. Hachem, Andrea J. Mothe, Charles H. Tator

**Affiliations:** ^1^ Division of Genetics and Development, Krembil Neuroscience Centre, Toronto Western Hospital, University Health Network, Ontario, Canada.; ^2^ Department of Surgery, Division of Neurosurgery, University of Toronto, Ontario, Canada.

**Keywords:** antioxidants, cell culture, growth factor, neural stem cells, oxidative stress

## Abstract

Transplantation of neural stem/progenitor cells (NSPCs) is a promising strategy in spinal cord injury (SCI). However, poor survival of transplanted stem cells remains a major limitation of this therapy due to the hostile environment of the injured cord. Oxidative stress is a hallmark in the pathogenesis of SCI; however, its effects on NSPCs from the adult spinal cord have yet to be examined. We therefore developed *in vitro* models of mild and severe oxidative stress of adult spinal cord–derived NSPCs and used these models to examine potential cell survival factors. NSPCs harvested from the adult rat spinal cord were treated with hydrogen peroxide (H_2_O_2_) *in vitro* to induce oxidative stress. A mild 4 h exposure to H_2_O_2_ (500 μM) significantly increased the level of intracellular reactive oxygen species with minimal effect on viability. In contrast, 24 h of oxidative stress led to a marked reduction in cell survival. Pretreatment with brain-derived neurotrophic factor (BDNF) for 48 h attenuated the increase in intracellular reactive oxygen species and enhanced survival. This survival effect was associated with a significant reduction in the number of apoptotic cells and a significant increase in the activity of the antioxidant enzymes glutathione reductase and superoxide dismutase. BDNF treatment had no effect on NSPC differentiation or proliferation. In contrast, cyclosporin A and thyrotropin-releasing hormone had minimal or no effect on NSPC survival. Thus, these models of *in vitro* oxidative stress may be useful for screening neuroprotective factors administered prior to transplantation to enhance survival of stem cell transplants.

## Introduction

Stem cell–based therapies have shown promising therapeutic potential in spinal cord injury (SCI).^1,2^ Transplantation of various stem cells including neural stem/progenitor cells (NSPCs), embryonic stem cells (ESCs), mesenchymal stem cells, and induced pluripotent cells ameliorated damage to the injured spinal cord in experimental models.^1,3^ The ability of adult NSPCs to self-renew yet remain committed to the neural lineage^4^ makes them particularly advantageous over other cell types, as they have no tumorigenic potential and also avoid many of the ethical issues associated with embryonic or fetal stem cells. Transplantation of NSPCs into spinal cord injured rodents has been shown to increase tissue sparing, reduce cavity size, secrete beneficial trophic factors and improve functional recovery.^5–8^ Despite these advances, poor survival of transplanted stem cells remains a major limitation of this therapy.^5,9^

The factors that contribute to the death of transplanted NSPCs remain unclear. Adult NSPCs seem to have robust mechanisms to withstand some of the hallmarks of the secondary injury in SCI including glutamate toxicity^10,11^ and hypoxia.^12^ However, their susceptibility to the elevated levels of reactive oxygen species (ROS) associated with SCI has not been fully examined. Oxidative stress plays a significant role in the pathogenesis of SCI.^13,14^ Shortly after the initial physical trauma, there is a marked increase in the production of ROS including superoxide, hydroxyl radicals, and hydrogen peroxide.^15,16^ High levels of ROS can lead to lipid peroxidation, oxidative chain reactions, and damage to cellular macromolecules leading to death of host neurons and glia.^17^ While low, nontoxic levels of ROS seem to maintain the proliferative capacity of neural stem cells and modulate their differentiation potential,^18,19^ higher levels such as those found in the injured spinal cord may lead to cell death. Indeed, *in vitro* studies have found that ESCs,^20^ fetal brain, or spinal cord–derived neural stem cells^21–24^ and adult bone marrow–derived mesenchymal stem cells^25^ are susceptible to hydrogen peroxide (H_2_O_2_)-induced oxidative stress. However, it is unclear whether adult NSPCs are equally susceptible to oxidative stress as there are no reports that thoroughly examine this issue.

We developed *in vitro* models of mild and severe oxidative stress applied to adult spinal cord–derived NSPCs using H_2_O_2_ and analyzed specific outcome measures of cellular stress and viability. Mild oxidative stress was induced after a 4 h exposure to H_2_O_2_ and was characterized by an increase in intracellular ROS with a minimal reduction in cell viability. Severe oxidative stress occurred after 24 h of H_2_O_2_ exposure and was associated with extensive cell death and membrane destruction. In contrast to H_2_O_2_-induced cell death, there was no effect of glutamate exposure on NSPC viability even at very high concentrations. With the H_2_O_2_ models, we tested three potential survival factors for their ability to protect against oxidative stress *in vitro*: cyclosporin A (CsA), which has been shown to increase survival of NSPCs *in vitro* and *in vivo*,^26^ brain-derived neurotrophic factor (BDNF) for its known survival effects in the nervous system,^27^ and thyrotropin-releasing hormone (TRH), as it has shown positive results in both experimental and human studies of SCI.^28,29^ We show for the first time that pretreatment with BDNF significantly increased NSPC survival *in vitro* following oxidative stress, as indicated by reduced intracellular ROS accumulation under mild stress and increased cell viability under severe stress. Furthermore, we show that this survival effect is mediated by a reduction in the number of apoptotic NSPCs and an increase in their antioxidant enzyme activity.

## Methods

### NSPC isolation and culturing

Cryogenically preserved NSPCs previously isolated from the central canal region of the spinal cord of transgenic adult female Wistar rats expressing green fluorescent protein were used in this study. The methods of isolation were previously described by our laboratory.^30,31^ After thawing, the frozen cells were grown as free floating neurospheres in growth media containing neurobasal media (Gibco-Invitrogen), B27 neural supplement (Gibco-Invitrogen), 2 mM L-glutamine (Gibco-Invitrogen), 100 μg/mL penicillin-streptomycin (Gibco-Invitrogen), 20 ng/mL epidermal growth factor (EGF, Sigma), 20 ng/mL basic fibroblast growth factor (FGF, Sigma), and 2 μg/mL heparin (Sigma). NSPCs were passaged every 7 days and cells between passages 3 to 6 were used for experiments.

NSPCs (20,000 cells/well) were dissociated using Accutase (Gibco-Invitrogen) and seeded on Matrigel (BD Biosciences Inc.) coated 24-well plates (Nunc) containing growth media. For the H_2_O_2_ studies, 30% H_2_O_2_ (Fisher Scientific) was added to the well to reach a final concentration ranging between 0 and 500 μM H_2_O_2_. For the glutamate studies, l-glutamate (Sigma) was dissolved in water then added to the wells in a final concentration ranging between 0 and 1 mM.

For the neuroprotection studies, NSPCs were pre-treated with various concentrations of CsA (BioShop Canada), BDNF (Peprotech), or TRH (Sigma) for 48 h (the factors were prepared in the growth media described above). Control cells were pretreated with growth media alone for 48 h. Then, H_2_O_2_ was added to each well to reach a final concentration of 500 μM. No washout was performed between factor treatment and H_2_O_2_ exposure.

### Dihydroethidium staining to show ROS

The accumulation of intracellular ROS in NSPCs was detected using dihydroethidium (DHE) (Gibco-Invitrogen). After a 4 h exposure to H_2_O_2_, NSPCs were incubated with 2.5 μM DHE (diluted in Hank's balanced salt solution containing Ca and Mg) for 10 min at room temperature then imaged using a Nikon Eclipse TE 300 microscope. Five random images were taken for each well at 10× magnification. DHE intensity was analyzed above a set threshold using Image J Software.

### Calcein staining to show live cells

Cell viability was assessed using calcein-AM dye (Gibco-Invitrogen). NSPCs were incubated with 5 μM calcein-AM (diluted in Hank's balanced salt solution containing Ca and Mg) for 10 min at room temperature then imaged using a Nikon Eclipse TE 300 microscope. Five random images were taken for each well at 10× magnification. The number of calcein+ cells was counted in each image to assess cell survival. A minimum of 750 cells per condition were counted.

### Lactate dehydrogenase assay to show membrane disruption and cell death

Cell death was assessed using the lactate dehydrogenase assay (Roche), which is a measure of impaired membrane integrity. Two hundred microliters of supernatant was removed from each well, centrifuged, and 100 μL of the remaining supernatant was placed in 96-well plates (BD Biosciences Inc.). One hundred microliters of assay mix was added to each well and incubated for 30 min at room temperature. Absorbance was measured at 490 nm using an ultraviolet plate reader. All values were normalized to control conditions.

### Immunostaining for cell differentiation and proliferation

Cell differentiation and proliferation were assessed using immunocytochemical staining, as we have previously described.^30^ After a 48 h treatment with the various factors, cells were fixed with 4% paraformaldehyde for 20 min at room temperature and washed with 0.1 M phosphate buffered saline (PBS). Cells were then blocked with 10% normal goat serum with 0.3% Triton-X 100 and 1.5% bovine serum albumin (depending on the primary antibody) for 1 h at room temperature. Afterward, cells were incubated with the primary antibody overnight at 4°C. The following primary antibodies were used: Ki67 (1:100; Novocastra Laboratories) for proliferating cells, nestin (1:2000; Millipore) for neural stem/progenitor cells, GFAP (1:2000; Dako, Burlington, ON) for astrocytes, RIP (1:3; Developmental Studies Hybridoma Bank) for oligodendrocytes, βIII-tubulin (1:2000; Covance) for neuronal progenitor cells. Cells were then washed with 0.1M PBS, incubated with fluorescent Alexa 568 secondary antibody (1:500; Invitrogen) for 1 h, and washed with PBS. To counterstain for cell nuclei, cells were incubated in Hoechst and washed with PBS. Immunocytochemical staining was imaged using a Nikon Eclipse TE 300 microscope. For Ki67 staining, five random images were taken for each well at 10× magnification and the percentage of Ki67+ cells was quantified per image. For all other antibodies, 10 random images were taken for each well at 20× magnification and the percentage of positive cells was quantified per image.

### Terminal deoxynucleotidyl transferase-mediated dUTP nick-end labeling staining and morphological analysis to measure apoptosis

Cell apoptosis was assessed using terminal deoxynucleotidyl transferase-mediated dUTP nick-end labeling (TUNEL) staining. Following each treatment condition, NSPCs were fixed with 4% paraformaldehyde for 20 min at room temperature and washed with 0.1M PBS. TUNEL staining was done according to the manufacturer's instructions (Roche). To counterstain for cell nuclei, cells were incubated in Hoechst and washed with PBS. TUNEL-positive cells were visualized using a Nikon Eclipse TE 300 microscope. Five random images were taken for each well at 10× magnification and the percentage of TUNEL+ cells was quantified per image. Apoptotic morphology of cells was visualized using bright field microscopy.

### Measurement of antioxidant enzyme activity

Following each treatment condition, NSPCs were detached from plates using 1mM ethylenediaminetetraacetic acid. Cells were then centrifuged (1500 *g* for 10 min at 4°C), lysed, and centrifuged at 10,000 *g* for 15 min at 4°C. The supernatants were used for all subsequent experiments. The activity of antioxidant enzymes glutathione reductase (GR) and superoxide dismutase (SOD) was assessed using commercially available kits according to the manufacturer's instructions (Cayman Chemicals).

### Statistical analysis

All data are presented as mean±standard error of the mean and were analyzed using SigmaStat 3.1 software. Statistical differences between multiple groups were assessed using one-way analysis of variance and Bonferroni's or Dunnett's post-hoc corrections. Differences between two groups were assessed using a two-tailed Student's *t*-test. A *p* value of <0.05 was set as the significance level for all tests. All experiments were performed in triplicate wells and repeated at least three times.

## Results

### Induction of mild oxidative stress in adult NSPCs

**FIG. 1.**
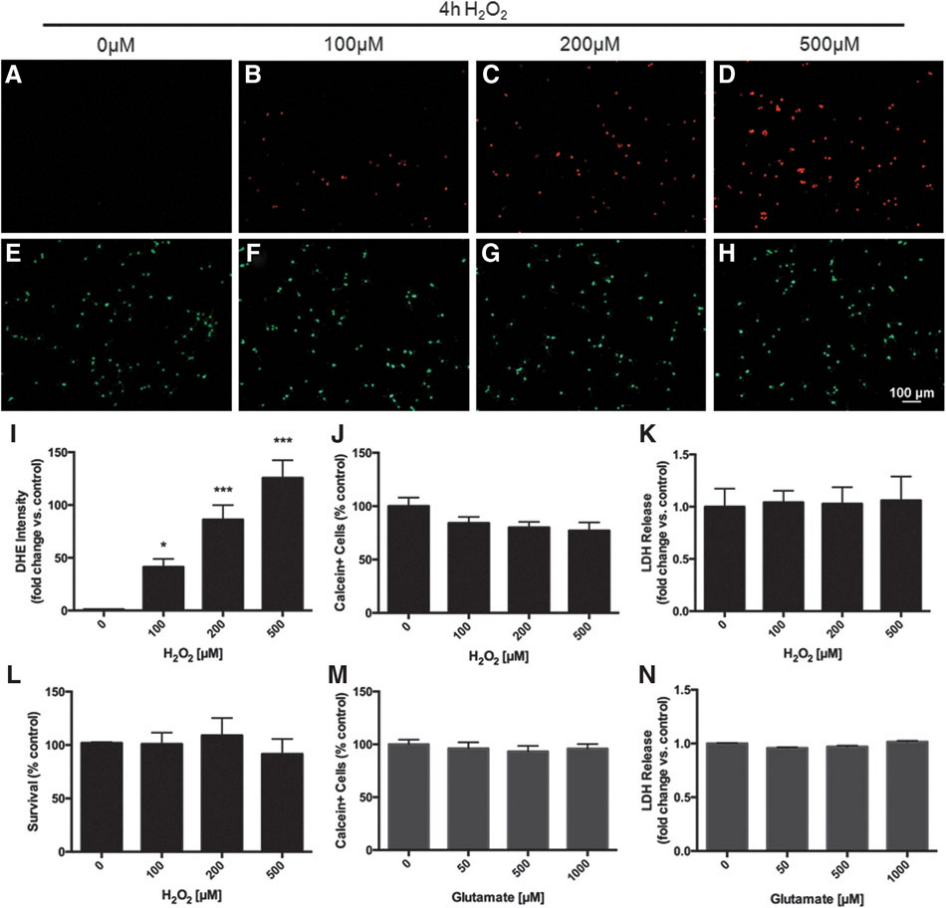
Mild oxidative stress: A 4 h exposure to hydrogen peroxide (H_2_O_2_) increases intracellular reactive oxygen species (ROS) in neural stem/progenitor cells (NSPCs) with minimal effect on cell viability. After a 4 h exposure to increasing concentrations of H_2_O_2_ (0–500μM), NSPCs displayed a significant accumulation of intracellular ROS as demonstrated by increased dihydroethidium (DHE) staining intensity **(A–D)**. At the highest concentration tested (500 μM), there was a 125-fold increase in DHE intensity compared with control (*p*<0.001) **(I)**. The number of live cells (calcein+) was reduced to 77.1±7.8% of control although this did not reach significance **(E–H**, **J)**. Membrane integrity was not impaired as there was no change in lactate dehydrogenase (LDH) release **(K**, **L)**. Glutamate treatment for 4 h displayed no change in NSPC survival **(M**, **N)**. Data are expressed relative to control (0 μM H_2_O_2_ or 0 μM glutamate). Asterisks indicate a statistically significant difference compared with control: *p<0.05, ***p<0.001.

To investigate the effects of H_2_O_2_ on adult rat spinal cord NSPCs *in vitro*, we cultured cells in various concentrations of H_2_O_2_ (0–500 μM) and assessed intracellular ROS levels and cell viability at different time points. A mild oxidative stress was induced after a 4 h exposure to H_2_O_2_. NSPCs showed a significant increase in intracellular ROS (*p*<0.05) as indicated by increased DHE intensity (Fig. 1 A–D, I). Cell viability was only minimally reduced at the highest concentration of H_2_O_2_ (500 μM), and membrane integrity was maintained across all concentrations (Fig. 1 E–H, J–L). In comparison, a 4h exposure to glutamate (0–1000 μM) did not exert any effects on intracellular ROS (data not shown), cell viability (Fig. 1 M) or membrane integrity (Fig. 1 N). In contrast, a 20 min exposure to 500μM of glutamate was shown to cause significant death in neuronal cultures *in vitro*.^32^

### Induction of severe oxidative stress in adult NSPCs

**FIG. 2.**
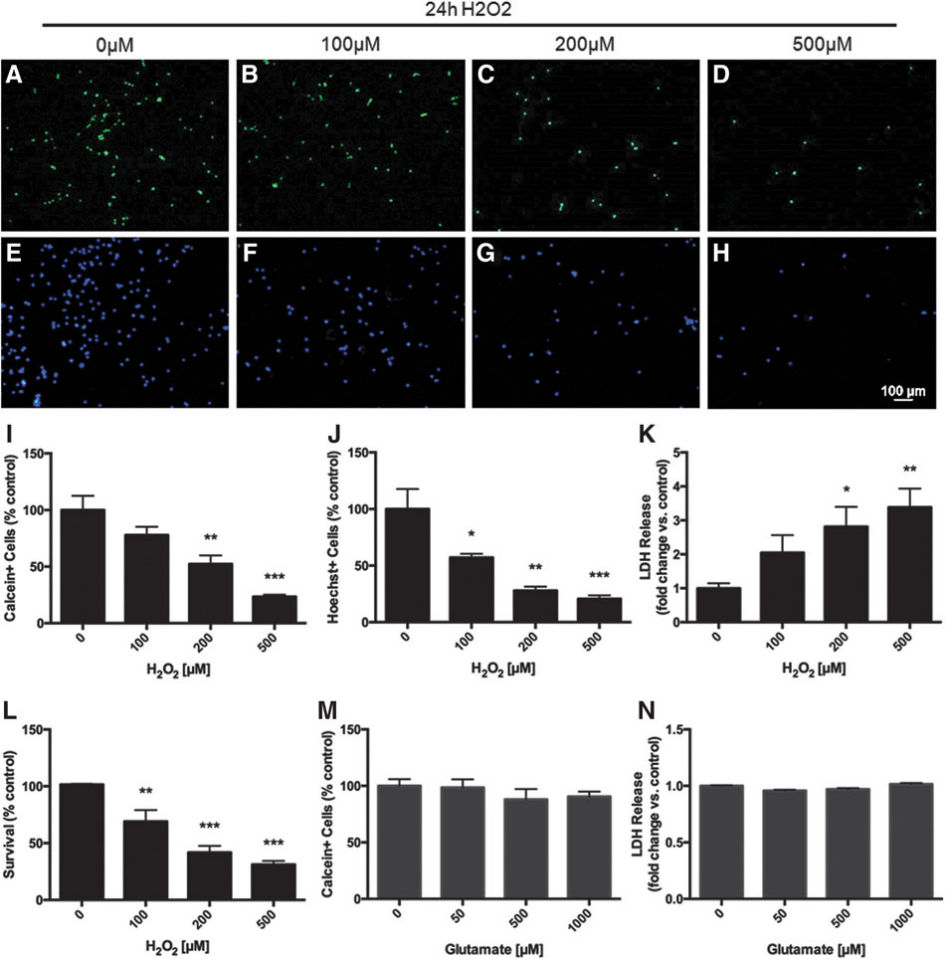
Severe oxidative stress: A 24 h exposure to H_2_O_2_ decreases NSPC viability. NSPCs treated with increasing concentrations of H_2_O_2_ (0–500 μM) for 24 h displayed a dose-dependent decrease in the number of live cells as measured by both calcein **(A–D**, **I)** and Hoechst **(E–H**, **J)** staining. Membrane integrity, assessed via the LDH assay, was also significantly impaired following H_2_O_2_ exposure for 24 h **(K**, **L)**. Estimates of cell viability were similar between all three tests: calcein staining, Hoechst staining, and LDH release indicated cell survival after 24 h of 500 μM H_2_O_2_ to be 23.4±1.7%, 20.7±3.02%, and 31.2±3.1% of control, respectively (*p*<0.001). In contrast, 24 h exposure to glutamate was not toxic to NSPCs, as calcein+ cells and LDH release were similar to control at all concentrations **(M**, **N)**. Data are expressed relative to control (0 μM H_2_O_2_ or 0 μM glutamate). Asterisks indicate a statistically significant difference compared with control: *p<0.05, **p<0.01, ***p<0.001.

To induce a more severe oxidative stress, we extended the exposure time to 24 h H_2_O_2_, at which point NSPCs displayed a dose-dependent decrease in the number of live cells as measured by calcein staining (Fig. 2 A–D, I). At the highest concentration of H_2_O_2_ (500 μM), cell survival was reduced to only 23.4±1.7% of control (*p*<0.001). Live cell numbers based on calcein staining was corroborated by staining with Hoechst nuclear dye (Fig. 2 E–H, J). Moreover, there was a marked decrease in the proliferative capacity of surviving NSPCs exposed to even the lowest concentration of H_2_O_2_ (100 μM) for 24 h, as indicated by decreased Ki67 staining (data not shown). Membrane integrity was also significantly impaired at this time as assessed by the lactate dehydrogenase (LDH) assay (Fig. 2 K, L). At the highest concentration of H_2_O_2_ (500 μM) there was a 3.4-fold increase in LDH release relative to control (*p*<0.001). LDH release data converted to percent survival showed a remarkably similar estimate of cell viability compared with the percentage of calcein+ and Hoechst+ cells (Fig. 2L). In contrast, a 24 h glutamate exposure did not produce any significant changes in cell viability even at 1mM concentration (Fig. 2M, N). Based on these findings, 500 μM H_2_O_2_ was selected as the optimal concentration to model oxidative stress in NSPCs and was thus used in subsequent experiments.

### BDNF pretreatment attenuates cellular injury in mild and severe models of oxidative stress

We next used these models of mild and severe oxidative stress to test the ability of BDNF, CsA, and TRH to protect NSPCs against H_2_O_2_-induced cell death. NSPCs were treated with various concentrations of either CsA, BDNF, or TRH for 48 h followed by exposure to 500 μM H_2_O_2_.

**FIG. 3.**
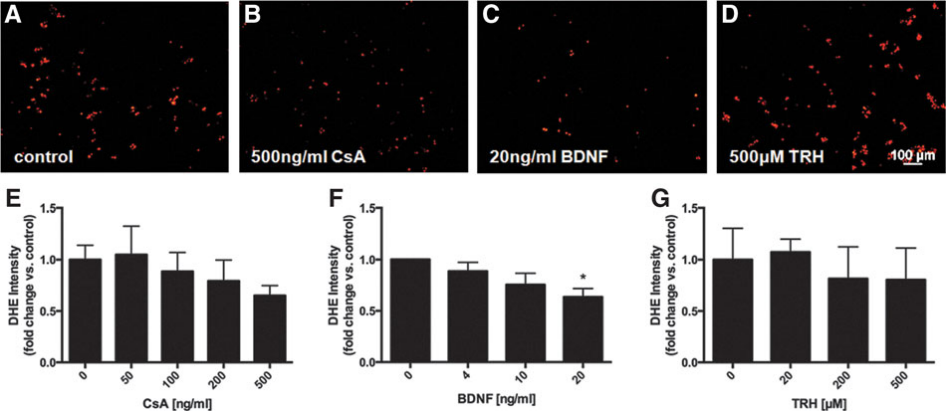
Effects of factor pretreatment on intracellular ROS accumulation after mild oxidative stress in NSPCs. Representative images of DHE staining to measure intracellular ROS **(A–D)**. Pretreatment with 500 ng/mL cyclosporin A (CsA) for 48 h decreased intracellular ROS following a 4 h exposure to 500 μM H_2_O_2_ as measured by DHE intensity **(B**, **E)**, although the reduction was not significant (*p*>0.05). Pretreatment with 20 ng/mL brain-derived neurotrophic factor (BDNF) significantly decreased intracellular ROS (*p*<0.05) **(C**, **F)**. Thyrotropin releasing hormone (TRH) had no effect at even the highest concentration tested **(D**, **G)**. Data are expressed relative to control (no factor treatment). Asterisks indicate a statistically significant difference compared with control: *p<0.05.

**FIG. 4.**
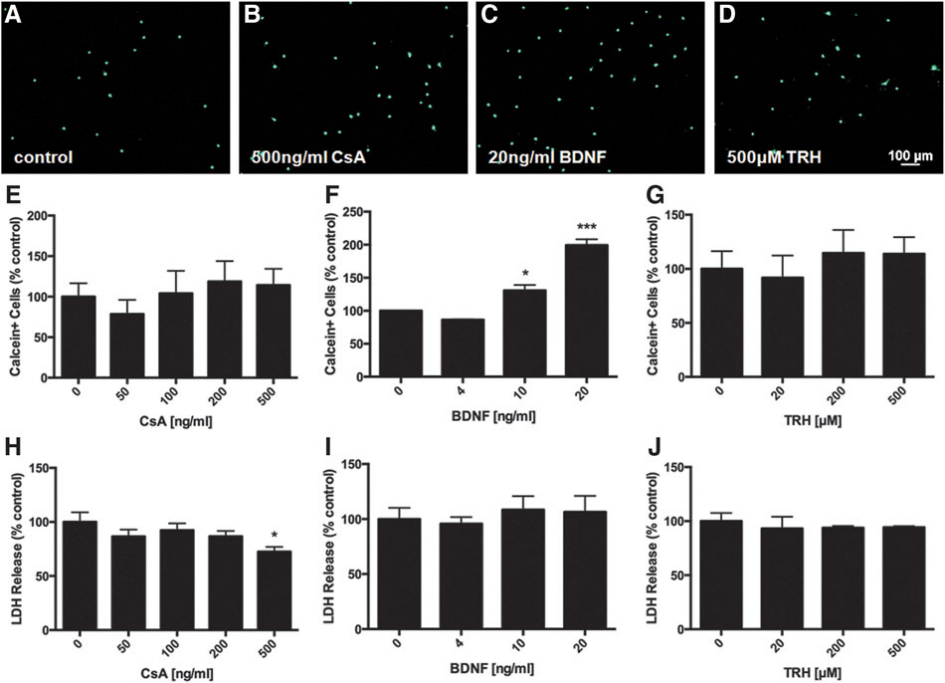
Effects of factor pretreatment on cell viability after severe oxidative stress in NSPCs. Representative images of calcein staining to measure cell viability **(A–D)**. Pretreatment with 20 ng/mL BDNF **(C**, **F)** for 48 h significantly increased the number of live cells relative to control by 199.2±9.0% (*p*<0.001) after a 24 h exposure to 500 μM H_2_O_2_. CsA **(B**, **E)** and TRH **(D**, **G)** had no significant effect at any concentration. 500ng/mL CsA pretreatment significantly decreased LDH release to 72.4±4.6% of control (*p*<0.05) **(H)**, whereas BDNF **(I)** and TRH **(J)** had no effect. Data are expressed relative to control (no factor treatment). Asterisks indicate a statistically significant difference compared with control: *p<0.05, ***p<0.001.

Pretreatment with BDNF (20 ng/mL) attenuated the increase in intracellular ROS induced by 4 h of oxidative stress (*p*<0.05). CsA (500 ng/mL) showed a trend towards decreased ROS while TRH had no effect at any concentration (Fig. 3). BDNF pretreatment was also able to protect NSPCs from severe oxidative stress. After 24 h of H_2_O_2_ exposure, NSPCs pretreated with 20 ng/mL BDNF had an approximately 2-fold increase in the number of surviving cells compared with controls (*p*<0.001) (Fig. 4 C, F). Pretreatment with 500 ng/mL CsA led to a slight increase in the number of surviving cells, although this did not reach significance, (Fig. 4 B, E) and a 27.6% reduction in LDH release compared with control (*p*<0.05) (Fig. 4 H).

### BDNF pretreatment does not alter NSPC phenotype or proliferation

**FIG. 5.**
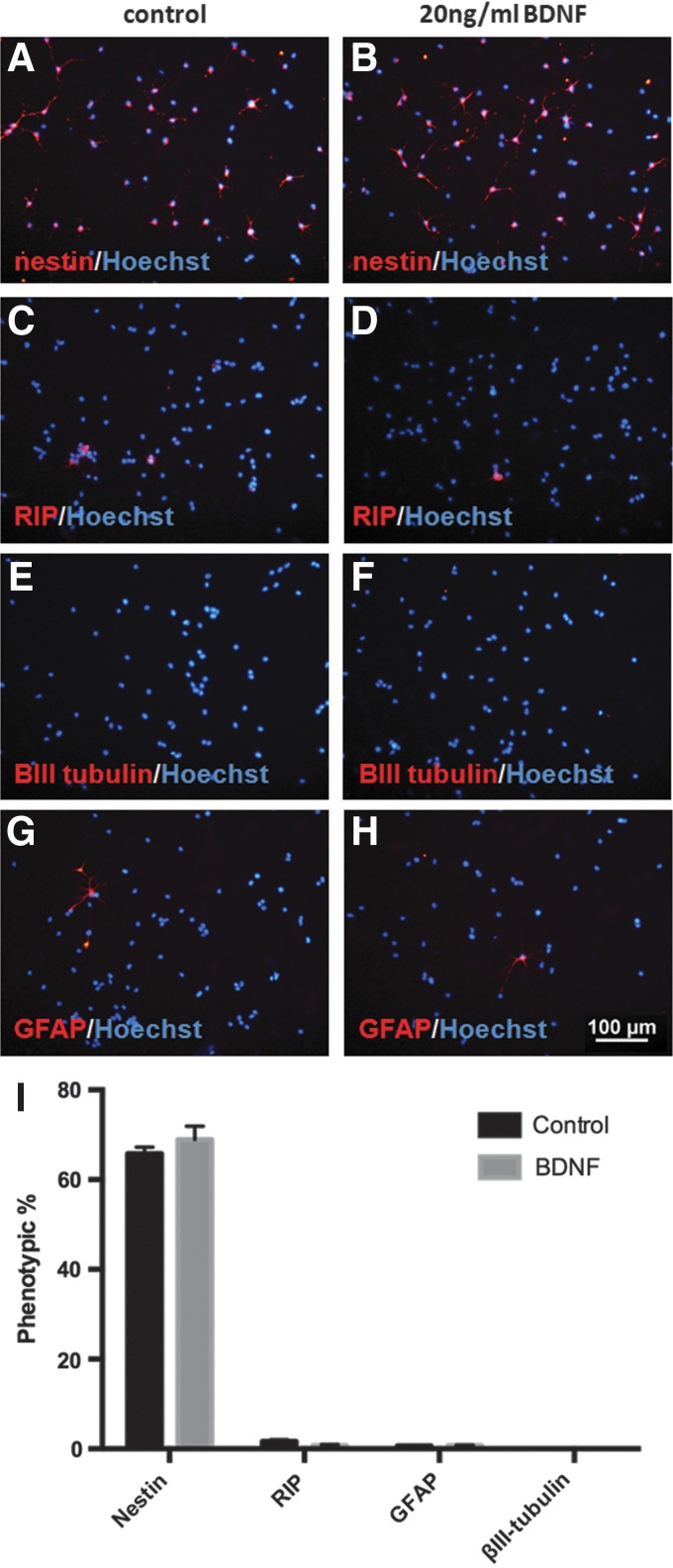
BDNF pretreatment does not alter the cellular phenotype of NSPCs. Treatment with 20 ng/mL BDNF for 48 h did not alter the differentiation profile of NSPCs. Representative images of nestin **(A**, **B)**, RIP **(C**, **D)**, βIII-tubulin **(E**, **F)**, and GFAP **(G**, **H)** staining (red) counterstained with Hoechst (blue). The majority of cells were nestin-positive (65.7±1.5% in the control versus 68.9±3.0%in the BDNF group) and very few cells expressed markers for RIP, GFAP, or βIII-tubulin **(I)**.

To better understand the mechanisms underlying the neuroprotective effects of BDNF pretreatment on NSPCs exposed to oxidative stress, we first examined if BDNF induced any changes in NSPC differentiation. We found no difference in the cellular phenotype of NSPCs following a 48 h BDNF pretreatment compared with control. The majority of cells in both groups remained as undifferentiated stem cells (nestin+) and expressed low levels of oligodendrocyte (RIP), neuron βIII-tubulin, and astrocyte (GFAP) markers (Fig. 5).

**FIG. 6.**
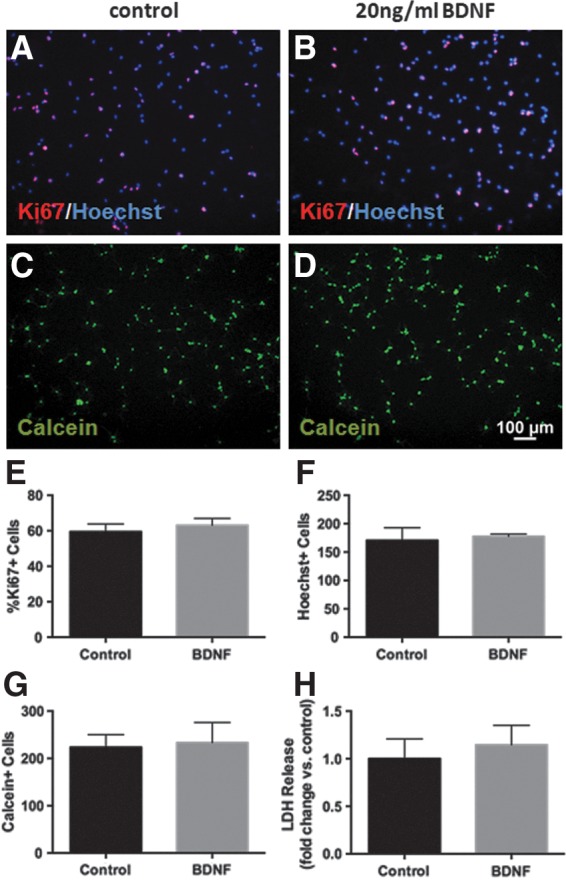
BDNF pretreatment does not induce proliferation of NSPCs. Representative images of Ki67 staining (red) counterstained with Hoechst (blue) after 48 h of control **(A)** and 20 ng/mL BDNF **(B)** treatment. Treatment with 20 ng/mL BDNF for 48 h did not alter proliferation in NSPCs as measured by Ki67 staining **(E)**, nor did it change the total number of cells as measured by Hoechst staining **(F)**. There was also no change in the survival of cells after 48 h of BDNF treatment as measured by calcein staining **(C**, **D**, **G)** or LDH release **(H)**.

Next, we assessed whether the increased number of surviving cells in the BDNF pretreatment group was influenced by any proliferative effects this factor may have on NSPCs. Following a 48 h treatment with 20 ng/mL BDNF, we stained cells using the proliferative marker Ki67 and found that BDNF did not alter the percentage of Ki67+cells compared with control (Fig. 6 A, B, E) nor did it affect the total number of cells as measured by Hoechst staining (Fig. 5 F). Furthermore, 48h of BDNF treatment did not alter live cell numbers or membrane integrity compared with control (Fig.6 G, H).

### BDNF pretreatment reduces H_2_O_2_-induced cellular apoptosis in NSPCs

**FIG. 7.**
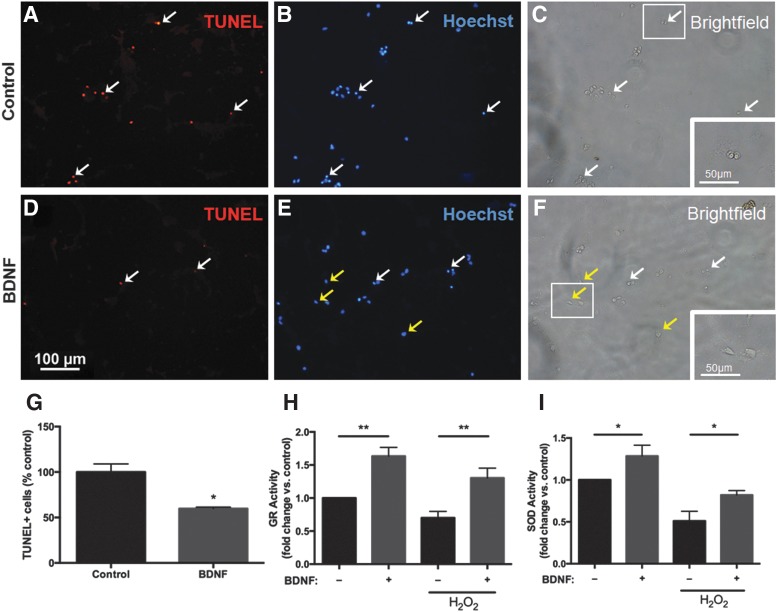
BDNF pretreatment decreases the number of apoptotic cells and increases antioxidant activity in NSPCs exposed to oxidative stress. Representative images of double-labeled terminal deoxynucleotidyl transferase-mediated dUTP nick-end labeling (TUNEL) staining **(A**, **D)** and Hoechst nuclear marker **(B**, **E)** with corresponding bright field images **(C**, **F)** after 48 h factor pretreatment followed by 4 h of H_2_O_2_ exposure. White arrows indicate TUNEL+ cells with characteristic apoptotic morphology on Hoechst and bright field imaging. Following H_2_O_2_ exposure, many cells under control conditions were TUNEL+ **(A**, *white arrows*), with highly condensed nuclei **(B**, *white arrows*), and rounded up nuclei and cell bodies **(C**, *white arrows*). There were significantly fewer TUNEL+ cells **(D**, *white arrows*), with apoptotic morphology **(E**, **F**, *white arrows*) seen among BDNF pretreated cells. The majority of BDNF pretreated cells displayed normal nuclear **(E**, *yellow arrows*) and cellular **(F**, *yellow arrows*) morphology. Insets represent a higher magnification of the boxed areas **(C**, **F)**. Based on quantitative cell counts, BDNF pretreatment significantly reduced the number of TUNEL+ cells following exposure to 4 h of H_2_O_2_ compared with control **(G)**. BDNF treatment increased cellular glutathione reductase (GR) and superoxide dismutase (SOD) activity compared with control (*p*<0.05). H_2_O_2_ exposure reduced the activity of both enzymes in control cells, however, cells pretreated with BDNF maintained a higher level of GR (*p*<0.01) and SOD (*p*<0.05) activity **(H**, **I)**. Data are expressed relative to control (no BDNF treatment, no H_2_O_2_ exposure). Asterisks indicate a statistically significant difference compared with control: *p<0.05, **p<0.01.

Following induction of oxidative stress, there were significantly fewer TUNEL+ cells in the BDNF pretreatment group compared with control (Fig. 7A, D, G). TUNEL+ cells displayed characteristic apoptotic morphology including condensed nuclei (Fig. 7B, white arrows) and rounded up cell bodies (Fig. 7C, white arrows). These apoptotic features were markedly reduced in BDNF pretreated cells (Fig. 7E, F).

### BDNF pretreatment enhances antioxidant enzyme activity in NSPCs

In addition to its antiapoptotic role, we wanted to determine if BDNF directly targets antioxidant pathways. To test this we examined the activity of the antioxidant enzymes glutathione reductase (GR) and superoxide dismutase (SOD) (Fig. 7H, I). Prior to H_2_O_2_ exposure, 48 h BDNF pretreatment significantly increased the activity of GR (1.6-fold increase relative to untreated cells, *p*<0.01) and SOD (1.3-fold increase relative to untreated cells, *p*<0.05). Following H_2_O_2_ exposure, there was a significant decrease in GR and SOD activity in untreated cells; however, the activity of both enzymes remained elevated in cells pretreated with BDNF (*p*<0.05 vs. control+H_2_O_2_).

## Discussion

While increased production of ROS is a key component of the pathophysiology of SCI, its effects on adult NSPCs remains largely unknown. In this paper we show for the first time that H_2_O_2_ induces oxidative stress in adult rat spinal cord–derived NSPCs. Moreover, we were able to model this response *in vitro* with two injury levels—a mild stress associated with an accumulation of intracellular ROS but relatively high cell viability and a severe stress associated with significant incidence of cell death and loss of membrane integrity. There was consistency in our measures of cellular stress and viability, and therefore this H_2_O_2_ response test may be used as an effective *in vitro* model of oxidative stress in NSPCs to aid in identifying potential cell survival factors. Further supporting the importance of targeting oxidative stress to enhance NSPC survival, we found that the excitotoxic agent glutamate did not impair NSPC viability even at very high concentrations. Brazel et al. reported a similar finding in perinatal brain–derived subventricular zone (SVZ) cells demonstrating that these cells are not only resistant to glutamate excitotoxicty but also proliferate in response to glutamate exposure.^11^ Therefore, while both glutamate excitotoxicity and oxidative stress are key components in the pathophysiology of SCI, it appears that of these two insults, only oxidative stress acts to impair NSPC viability.

Previous studies have indicated that neural stem cells have greater antioxidant defenses relative to neurons, as they must carefully regulate ROS status to maintain self-renewal.^33^ Indeed, nontoxic, low level increases in H_2_O_2_ exposure have been shown to induce proliferation of brain-derived neural stem cells^18^ and modulate the differentiation potential of adult NSPCs.^19^ However, higher levels of H_2_O_2_ induce oxidative stress in various stem cell lines leading to decreased proliferation, reduced membrane integrity, and eventual cell death.^20–24^ Thus, in the present study we focused on high levels of H_2_O_2_ to determine the susceptibility of NSPCs to oxidative stress as these conditions may be more reflective of the stresses seen in SCI. We found that 500 μM H_2_O_2_ significantly increased ROS levels in NSPCs after 4 h and decreased survival to approximately 20% after 24 h. While our findings suggest that adult NSPCs have a slightly lower susceptibility to oxidative stress compared with reports in embryonic or fetal stem cells,^20,21,23,24^ direct comparisons were not made and differences could be a result of culturing conditions and cell preparations.

Exogenous application of H_2_O_2_ can cause membrane depolarization and cytochrome c release from cells thus initiating an apoptotic cascade.^34^ We found that NSPCs began expressing apoptotic characteristics (TUNEL+ staining and apoptotic morphology) after 4 h of oxidative stress. However, increasing the magnitude of oxidative stress may lead to a transition from apoptosis to necrosis.^34^ Under our severe stress model, very few cells remained substrate adherent and thus TUNEL quantification could not be performed. However, of the cells that remained adherent, we saw morphological signs of both apoptosis (rounding up of nuclei and cell bodies, formation of apoptotic bodies) and necrosis (cell swelling and bursting). LDH release was also significantly increased at this time point suggesting decreased membrane integrity and progression toward necrosis.

Using the H_2_O_2_ models to test for potential survival factors we found that pretreatment with 20 ng/mL BDNF protected NSPCs from H_2_O_2_-induced cell death. While BDNF is known to play a significant role in central nervous system development, most studies have focused on BDNF as an agent to induce neuronal differentiation of stem cells^35^ and enhance the survival of mature neurons.^27^ However, the utility of BDNF as a survival factor for undifferentiated adult stem cells is less well understood. In addition, the effects of BDNF on adult spinal cord- derived NSPCs have not been fully examined. Here we present a BDNF treatment protocol that does not alter the phenotype of NSPCs, yet enhances their survival in the context of oxidative stress.

We found that a 48 h pretreatment with 20 ng/mL BDNF did not alter NSPC proliferation compared with control. Our results are consistent with previous reports demonstrating that the same concentration of BDNF has no effect on the proliferation of embryonic-derived spinal cord progenitor cells^36^ or brain-derived neural stem cells^37^ even after seven days of treatment. While other studies report increased proliferation of neural stem cells treated with BDNF in combination with EGF compared with EGF treatment alone, these studies utilized either higher concentrations of BDNF or an extended treatment period.^38,39^ The present study used a standard proliferative culture medium containing EGF and FGF2 which we have used in our previous studies and we have shown these conditions increase cell proliferation. Thus, 20 ng/mL BDNF in EGF/FGF2 culture does not increase NSPC proliferation.

We also found no effect of BDNF treatment on the differentiation profile of NSPCs. While BDNF has previously been used to induce neuronal differentiation of brain-derived fetal stem cells,^40^ embryonic-derived spinal cord progenitor cells,^36^ and SVZ cells^41^ *in vitro*, these protocols used high concentrations of BDNF (100 ng/mL) and longer treatment periods (7 days) compared with the current study. In fact, Arsenijevic and Weiss demonstrated that BDNF-induced neuronal differentiation of neural stem cells requires an exposure time greater than 48 h.^42^ Therefore, based on our findings, a low dose, short exposure to BDNF does not alter the phenotype of adult spinal cord–derived NSPCs but instead protects against oxidative stress.

To assess the mechanistic pathway of BDNF neuroprotection on NSPCs following oxidative stress, we examined changes in apoptotic properties and antioxidant enzymes. We found that BDNF pretreatment inhibited the proapoptotic effects of H_2_O_2_ on NSPCs as demonstrated by a reduction in TUNEL+ cells and apoptotic morphology. The antiapoptotic effects of BDNF on various differentiated neuronal cell populations are well described and involve PI3K/Akt and MAPK pathways.^43–45^ There are significantly fewer reports of BDNF-mediated neuroprotection in undifferentiated neural stem cells exposed to neurotoxic insults,^46–48^ although similar pathways appear to be involved. Here we present the first report describing the antiapoptotic effects of BDNF on adult spinal cord-derived NSPCs.

In addition to its antiapoptotic properties, we also found that BDNF may play a direct role in NSPC antioxidant defenses by enhancing the activity of antioxidant enzymes. Protection against oxidative stress relies on the activity of antioxidant enzymes like SOD, GR, and catalase in order to catalyze ROS degradation. After 48 h of BDNF treatment we saw a significant increase in the activity of GR compared with control. This effect was maintained in the context of oxidative stress as BDNF pretreatment prevented the decrease in GR activity induced by H_2_O_2_ exposure and effectively reduced the accumulation of intracellular ROS. GR plays a critical role in maintaining appropriate intracellular ROS levels through its involvement in the glutathione pathway. GR catalyzes the reduction of oxidized glutathione to reduced glutathione (GSH), which subsequently catalyzes H_2_O_2_ into H_2_O. In the context of oxidative stress where high levels of intracellular ROS accumulate in the cell, high GR activity is essential for the continual regeneration of GSH and degradation of H_2_O_2_.^49^ Our observations agree with previous studies that found BDNF increased the activity of glutathione reductase in primary neuronal cultures.^50,51^ In primary fetal hypothalamic neurons, BDNF treatment attenuated the reduction in GSH levels associated with ethanol-induced oxidative stress effectively reducing intracellular ROS.^52^

We also found that BDNF treatment attenuated the decrease in SOD activity induced by exposure to H_2_O_2_. BDNF has been shown to increase SOD levels in endothelial progenitor cells^53^ and hippocampal neurons,^51^ protecting against *in vitro* oxidative stress. Intrathecal administration of BDNF into spinal cord injured rodents also attenuated the down-regulation of copper/zinc SOD in neurons after acute SCI.^54^ SOD catalyzes the conversion of superoxide anion to H_2_O_2_ and is thus not directly involved in the degradation of H_2_O_2_. However, exogenous H_2_O_2_ treatment triggers a cascade of ROS generation including production of superoxide anions and thus enhanced SOD activity may be important in attenuating H_2_O_2_-mediated damage.^34^ Although we found that SOD activity was elevated following BDNF pretreatment, BDNF had a much more potent effect on enhancing GR activity. Therefore, the sustained up regulation of GR activity may be a major mechanism in BDNF-mediated protection against H_2_O_2_-induced oxidative stress.

Although BDNF pretreatment significantly enhanced the survival of NSPCs when exposed to oxidative stress, NSPCs treated with BDNF without administration of H_2_O_2_ did not show any change in cell survival compared with untreated cells. Therefore, we show that the BDNF treatment did not exert general neuroprotective effects and was in fact specific to protecting against oxidative stress.

The utility of a BDNF pretreatment approach to enhance NSPC survival in the context of oxidative stress is clinically advantageous since pretreatment avoids continual growth factor exposure which may exert negative physiological effects. Recently, pretreatment of embryonic-derived neural stem cells with BDNF has been shown to increase survival after transplantation in an experimental model of hypoxic–ischemic stroke.^55^ Therefore, in view of the *in vitro* findings presented here, BDNF pretreatment of adult spinal cord–derived NSPCs warrants further investigation examining its utility in an *in vivo* animal model of SCI.

In contrast to BDNF, we saw only a minimal protective effect with CsA pretreatment. CsA has been found to increase brain and spinal cord–derived NSPC survival^26,56^ and attenuate mitochondrial dysfunction and ROS toxicity in models of central nervous system (CNS) trauma.^57–59^ It is likely that the ability of CsA to protect NSPCs against H_2_O_2_-induced oxidative stress is linked to its role in blocking mitochondrial permeability transition pore formation upon exposure to ROS.^60^ While we saw only a modest protective effect with CsA pretreatment, it is possible that a longer exposure would have led to an enhanced effect.

Unlike CsA or BDNF, we did not find any survival effects of TRH in the context of oxidative stress. Our rationale for testing TRH as a potential neuroprotective factor stems from reports showing its ability to improve recovery in experimental and human studies of SCI^28,29^ likely through attenuating various components of the secondary injury. This is the first study to examine the effects of TRH on adult NSPCs. While our current findings suggest TRH does not significantly protect NSPCs against H_2_O_2_-induced oxidative stress, given the very short half-life of this compound, it is possible that repeated administration of TRH, as utilized in other studies, may be required to see any effect.^28,29^ Future studies may also examine the expression of TRH receptor on NSPCs to better understand the lack of responsiveness to TRH.

Much of the existing literature on neural stem cells is based on experiments involving primary cell cultures. To our knowledge, this is the first study examining oxidative stress in cryogenically preserved neural stem cells. Clinical translation of any stem cell-based therapy requires the preservation of cell stocks prior to transplantation, and thus, investigating the properties of cryogenically preserved cells is an important step towards clinical application. Based on preliminary findings we did not see a significant difference in the response of primary and cryogenically preserved NSPCs to either our mild or severe models of *in vitro* oxidative stress. Lower cell viability has been reported in cryogenically preserved ESCs compared with primary cultures; however, this effect was most prominent within 2 days of thawing cells, and cell viability was completely restored following 5 days in culture.^61^ In our protocols, cryogenic cells were cultured for at least five days after thawing in order to avoid this potential issue.

In summary, we have developed mild and severe *in vitro* models of oxidative stress in adult NSPCs that can be used to assess potential neuroprotective candidates prior to their testing *in vivo*. We demonstrated that BDNF pretreatment protects adult NSPCs against *in vitro* oxidative stress through both antiapoptotic and antioxidant mechanisms and may therefore be an effective strategy to examine in future work in order to enhance survival of transplanted NSPCs in CNS trauma.

## Abbreviations Used

BDNF: brain-derived neurotrophic factor
CNS: central nervous system
CsA: cyclosporin A
DHE: dihydroethidium
EGF: epidermal growth factor
ESCs: embryonic stem cells
FGF: fibroblast growth factor
GR: glutathione reductase
GSH: glutathione
H_2_O_2_: hydrogen peroxide
LDH: lactate dehydrogenase
NSPCs: neural stem/progenitor cells
PBS: phosphate buffered saline
ROS: reactive oxygen species
SCI: spinal cord injury
SOD: superoxide dismutase
SVZ: subventricular zone
TRH: thyrotropin-releasing hormone
TUNEL: terminal deoxynucleotidyl transferase-mediated dUTP nick-end labeling
